# Black candy-plug technique for a large false lumen in a patient with complicated type B aortic dissection

**DOI:** 10.1016/j.jvscit.2026.102212

**Published:** 2026-03-04

**Authors:** Yukihisa Ogawa, Shunsuke Kamei, Hidekazu Furuya, Ryuichi Tamimoto, Akiyoshi Yamamoto, Hiroyuki Nishi

**Affiliations:** a Department of Radiology, Tokai University Hachioji Hospital, Hachioji, Japan; b Department of Cardiovascular Surgery, Tokai University Hachioji Hospital, Hachioji, Japan

**Keywords:** Candy-plug, Coil-in-plug, Flap penetration, Glue embolization, Type B aortic dissection

## Abstract

Persistent false lumen (FL) perfusion after thoracic endovascular aortic repair for type B aortic dissection remains challenging, especially in large FLs. We present successful FL thrombosis using a modified version of the candy-plug—the “black candy-plug” technique. With no re-entry available, the FL was accessed by intravascular ultrasound-guided flap penetration. A modified Excluder aortic extender was deployed in the FL, reinforced with coil-in-plug, and the residual gap was sealed with n-butyl-2-cyanoacrylate, achieving complete FL occlusion. Follow-up computed tomography confirmed stable device position, total FL thrombosis, and favorable early remodeling. The black candy-plug technique offers a practical option for large FLs.

Type B aortic dissection with false lumen (FL) enlargement remains a major clinical issue.^1^^,^^2^ The candy-plug technique, first described in 2013 as a method for FL occlusion,^3^ has since been adopted using various commercially available stent grafts.^4^, ^5^, ^6^

However, incomplete occlusion or recanalization after candy-plug placement has been described, particularly in patients with large FLs.^7^^,^^8^

In this report, we describe a modified candy-plug, “the black candy plug” technique, named for the characteristic radiographic appearance created by dense coil packing and n-butyl-2-cyanoacrylate (NBCA) sealing, which appear dark (“black”) on fluoroscopy. This technique combines an Excluder aortic extender (Ex-cuff; W.L. Gore & Associates) with coil-in-plug technique and adjunctive NBCA sealing to enhance embolic durability.

## Case report

A 68-year-old female had previously undergone thoracic endovascular aortic repair (TEVAR) for acute type B aortic dissection extending from zone 3 to the terminal aorta with rapid FL enlargement and open abdominal aortic aneurysm repair with a bifurcated graft for a coexisting infrarenal abdominal aortic aneurysm involved in the dissection 1 month earlier.

**Fig 1:**
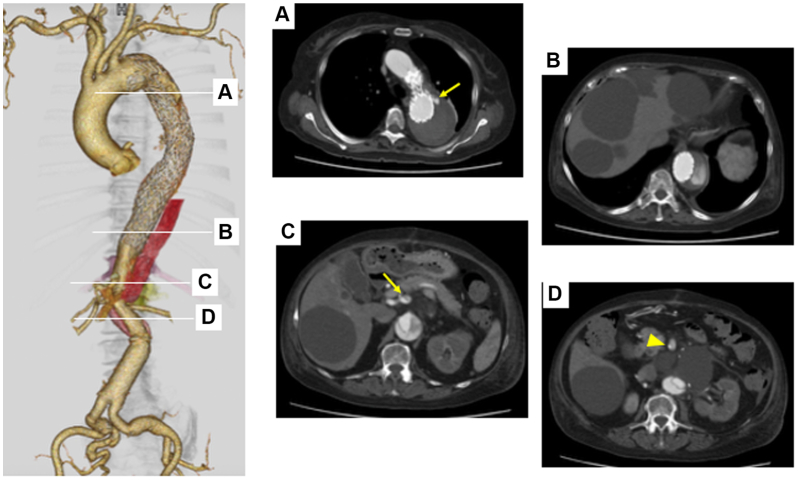
Contrast-enhanced computed tomography (CT) reveals progressive false lumen (FL) enlargement due to a type Ia endoleak after thoracic endovascular aortic repair (TEVAR) (**A**, *arrow*) and persistent retrograde flow **(B)** from multiple re-entries involving the celiacomesenteric trunk (**C,***arrow*; **D,***arrowhead*).

However, contrast-enhanced computed tomography (CT) revealed progressive FL enlargement from 54 mm to 68 mm over 1 month due to a type Ia endoleak after TEVAR (Fig 1, *A*) and persistent retrograde flow (Fig 1, *B*) from multiple re-entries involving the celiacomesenteric trunk (Fig 1, *C*). Additional endovascular treatment was therefore indicated.

A type Ia endoleak was managed with a one-debranching TEVAR combined with a left common carotid artery-left axillary artery bypass. To interrupt retrograde flow from the visceral reentries, placement of a candy plug in the FL was planned.

Because the patient had previously undergone Y-graft replacement with a single barrel anastomosis and no residual aortic re-entry was present, we elected to penetrate from the true lumen into the FL to obtain direct access.

**Fig 2:**
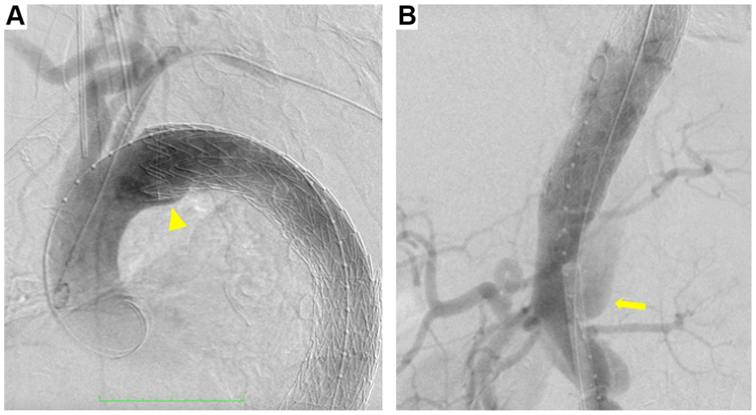
Aortography shows a proximal bird-beak configuration suggestive of a type Ia endoleak (**A,***arrowhead*), and retrograde flow at the level of celiacomesenteric trunk (**B,***arrow*), indicating distal false lumen (FL) reperfusion.

This procedure was performed in the operating room using a mobile C-arm fluoroscopy system (Cios Alpha VA30; Siemens Healthinees). After completing the carotid-axillary bypass, a 22F Dryseal sheath was inserted via the left femoral artery, an 8F sheath via the right femoral artery, and a 5F sheath via the left brachial artery. Aortography showed a proximal bird-beak configuration suggestive of a type Ia endoleak (Fig 2, *A*), and distal FL reperfusion at the level of the celiacomesenteric trunk (Fig 2, *B*). A 37 mm × 15 cm TAG Conformable stent graft (W.L. Gore & Associates) was then deployed just distal to the left common carotid artery.

**Fig 3:**
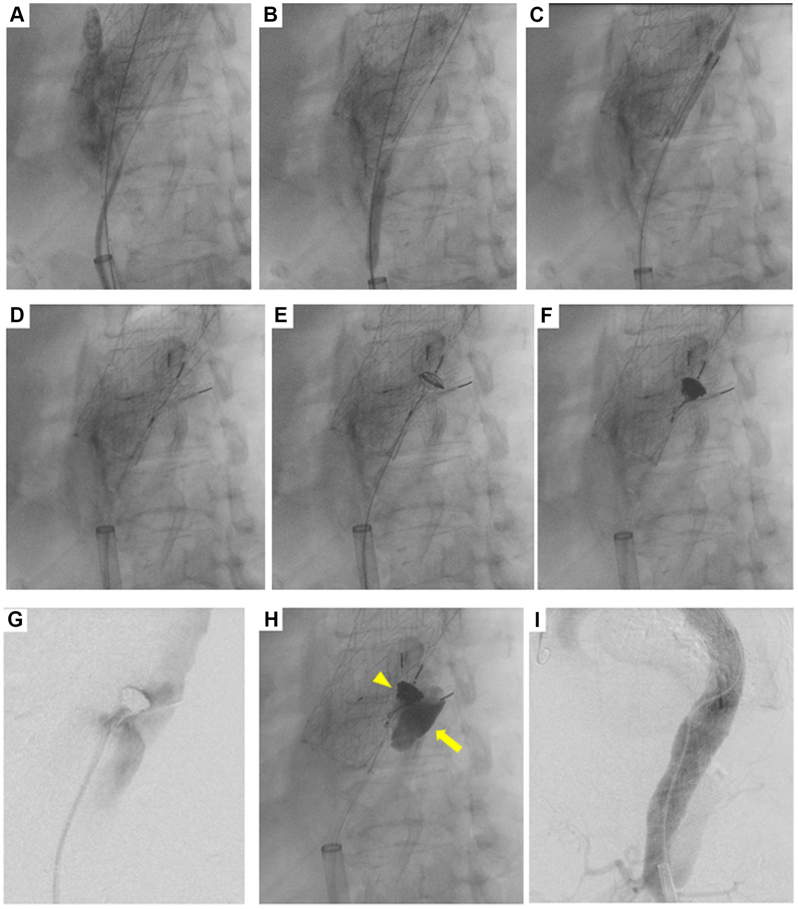
Under intravascular ultrasound (IVUS) guidance, the dissection flap was punctured from the true lumen into the false lumen (FL) approximately 2 cm distal to the caudal end of the thoracic stent graft, and a Radifocus guidewire was advanced into the FL **(A)**. The fenestration was then dilated using an 8 mm × 4 cm balloon catheter **(B)**. The modified Excluder aortic extender (Ex-cuff) was deployed at the distal level of the thoracic stent graft **(C)**. Subsequently, a 16-mm Amplatzer Vascular Plug I, preloaded with a 2.2F microcatheter was deployed at the waist of the modified Ex-cuff **(D)**. The microcatheter was then withdrawn into the plug, and four detachable coils were placed within the plug **(E** and **F)**. Angiography revealed persistent perigraft leakage around the Ex-cuff **(G)**. Therefore, 33% n-butyl-2-cyanoacrylate (NBCA) was slowly injected, achieving complete occlusion **(H)** (*arrowhead*, coil-in-plug; *arrow*, NBCA-Lipiodol). Completion angiography confirmed complete elimination of FL perfusion in the thoracic aortic segment **(I)**.

Through the right femoral approach, a guiding sheath (Parent Plus 60; Medikit Co) and intravascular ultrasound (IVUS; IntraSight Mobile; Volcano Corporation) were advanced, while a Rosch-Uchida needle was introduced from the left femoral sheath. Under IVUS guidance, the dissection flap was punctured from the true lumen into the FL approximately 2 cm distal to the caudal end of the thoracic stent graft, and a Radifocus guidewire was advanced into the FL (Fig 3, *A*). The fenestration was then dilated using an 8 mm × 4 cm balloon catheter (Mustang; Boston Scientific) (Fig 3, *B*).

To prepare the candy-plug, a 36 mm × 4.5 cm Ex-cuff (W.L. Gore & Associates) was placed on a side table. A durable polyester suture (2-0 Ethibond; Ethicon) was passed through the center of the Ex-cuff and tied circumferentially to create a diameter-reducing stitch, leaving approximately 10 mm of central clearance.^4^

This modified Ex-cuff was advanced over the guidewire into the FL and deployed at the distal level of the thoracic stent graft (Fig 3, *C*). Subsequently, a 16-mm Amplatzer Vascular Plug I (Abbott), preloaded with a 2.2F-microcatheter (Coiling Support, HI-LEX Co) was delivered through a 6F guiding catheter (Destination; Terumo Medical Co) and deployed at the waist of the modified Ex-cuff (Fig 3, *D*).^9^ The microcatheter was then withdrawn into the plug, and four 10 mm × 32 cm detachable coils (AZUR CX18; MicroVention, Inc) were placed within the plug to achieve complete intraplug thrombosis (Fig 3, *E* and *F*).

However, angiography revealed persistent perigraft leak around the Ex-cuff (Fig 3, *G*). To seal this gap, the microcatheter was redirected into the space between the plug and the FL wall, and 33% NBCA mixed with lipiodol was slowly injected while carefully avoiding reflux (Supplementary Video, online only), achieving complete occlusion (Fig 3, *H*). The fenestration through the dissection flap was intentionally left untreated.

Finally, coil embolization from the perigraft to the left subclavian artery was performed under the left subclavian artery occlusion using a 9-mm balloon catheter (Selecon MP, Medikit Co) via the left radial artery. Completion angiography confirmed complete elimination of FL flow in the thoracic aortic segment (Fig 3, I). The procedure ended without any complications.

**Fig 4:**
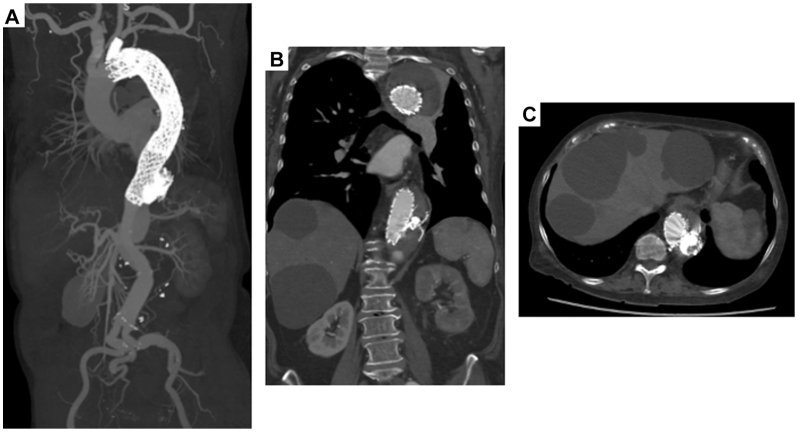
Contrast-enhanced computed tomography (CT) obtained at 3 weeks shows no migration or displacement of the candy-plug **(A)** and complete thrombosis of the thoracic false lumen (FL) **(B** and **C)**.

Contrast-enhanced CT obtained at 3 weeks demonstrated no migration or displacement of the candy-plug (Fig 4, *A*) and complete thrombosis of the thoracic FL (Fig 4, *B* and *C*).

At 3 months, noncontrast CT showed shrinkage of the FL, with the maximum short-axis aortic diameter from 67 mm to 53 mm, indicating favorable remodeling.

Informed consent was obtained for publication of this paper.

## Discussion

The black candy-plug technique represents the first reported application of a combined coil-in-plug and NBCA sealing within a candy-plug construct. This modification was developed to enhance the occlusive performance of the Ex-cuff-based candy-plug and provides a simple and cost-effective method for blocking retrograde flow even in large FLs.

Although several studies have reported the use of larger-diameter stent grafts as candy-plugs for large FLs, these devices require larger delivery sheaths and are more expensive.^5^^,^^6^ For these reasons, we prefer using the Ex-cuff as the candy-plug.

One of the major limitations of the Ex-cuff-based candy-plug is incomplete thrombosis or recanalization.^8^ First, inadequate occlusion of the Amplatzer Vascular Plug I may occur, particularly in patients with reduced coagulation capacity, such as those with rupture or on anticoagulation therapy.^9^ To overcome this issue, we reinforced intraplug thrombosis by deploying coils within the plug. This coil-in-plug technique has been described,^10^ but its application within a candy-plug has not previously been reported. The optimal number or volume of coils into the plug remains debatable; however, to maximize embolic volume, we prefer using hydrogel-coated coils.

A second limitation is candy-plug undersizing. When using an Ex-cuff, we generally select an oversizing of 10% to 20% relative to the mean FL diameter.^8^ In this case, the FL was partially thrombosed with an average patent lumen diameter of 29 mm (38 × 19 mm), thus a 36-mm Ex-cuff was chosen. However, the device was relatively undersized to the actual FL diameter, and a perigraft leak persisted.

We addressed this by sealing the gap with NBCA. Compared with coils, NBCA is inexpensive, provides immediate sealing, and effectively fills narrow channels. To avoid distal embolization, a high concentration (33%) was used; however, this choice depends heavily on operator experience and hemodynamic factors such as flow velocity and channel size.

This combined coil-in-plug and NBCA approach may also be beneficial in rupture cases, where coagulopathy is frequently present and conventional thrombosis-based occlusion may be insufficient.

Access into the FL is typically obtained through a re-entry tear; however, in cases without re-entries in the aorta or iliac arteries, penetration of the dissection flap has been reported.^11^ We adopted this approach in the present case. Because inadvertent vessel perforation is a potential complication, IVUS guidance is crucial to ensure correct puncture direction.

The fenestration created by flap penetration was intentionally left untreated to allow controlled decompression of the FL, as retrograde flow from visceral re-entries persisted. This strategy was intended to prevent excessive FL pressurization after candy-plug deployment. Further follow-up is needed to observe long-term FL remodeling.

## Conclusion

The black candy-plug technique enhances embolic stability and may provide a useful option in patients with large FLs where conventional approaches are inadequate.

## Funding

None.

## Disclosures

None

## Appendix (online only)

Supplementary Video (online only) Slow injection of 33% n-butyl-2-cyanoacrylate (NBCA; mixed with lipiodol at a 1:2 ratio; 4.5 mL) was performed under fluoroscopic guidance until the NBCA-lipiodol cast stabilized and completely filled the residual perigraft channel.
